# Hygiene and the world distribution of Alzheimer’s disease: Epidemiological evidence for a relationship between microbial environment and age-adjusted disease burden

**DOI:** 10.1093/emph/eot015

**Published:** 2013-07-11

**Authors:** Molly Fox, Leslie A. Knapp, Paul W. Andrews, Corey L. Fincher

**Affiliations:** ^1^Division of Biological Anthropology, Department of Anthropology and Archaeology, University of Cambridge, Pembroke Street, Cambridge CB2 3QY, UK, ^2^Department of Anthropology, University of Utah, 270 S 1400 E, Salt Lake City, UT 84112, USA, ^3^Department of Psychology, Neuroscience & Behaviour, McMaster University, 1280 Main Street W, Hamilton, ON L8S 4K1, Canada and ^4^Institute of Neuroscience and Psychology, University of Glasgow, 58 Hillhead Street, Glasgow G12 8QB, UK

**Keywords:** Alzheimer’s disease, hygiene hypothesis, inflammation, dementia, pathogen prevalence, Darwinian medicine

## Abstract

**Background and objectives:** Alzheimer’s disease (AD) shares certain etiological features with autoimmunity. Prevalence of autoimmunity varies between populations in accordance with variation in environmental microbial diversity. Exposure to microorganisms may improve individuals’ immunoregulation in ways that protect against autoimmunity, and we suggest that this may also be the case for AD. Here, we investigate whether differences in microbial diversity can explain patterns of age-adjusted AD rates between countries.

**Methodology:** We use regression models to test whether pathogen prevalence, as a proxy for microbial diversity, across 192 countries can explain a significant amount of the variation in age-standardized AD disability-adjusted life-year (DALY) rates. We also review and assess the relationship between pathogen prevalence and AD rates in different world populations.

**Results:** Based on our analyses, it appears that hygiene is positively associated with AD risk. Countries with greater degree of sanitation and lower degree of pathogen prevalence have higher age-adjusted AD DALY rates. Countries with greater degree of urbanization and wealth exhibit higher age-adjusted AD DALY rates.

**Conclusions and implications:** Variation in hygiene may partly explain global patterns in AD rates. Microorganism exposure may be inversely related to AD risk. These results may help predict AD burden in developing countries where microbial diversity is rapidly diminishing. Epidemiological forecasting is important for preparing for future healthcare needs and research prioritization.

## Abstract (precis)

People living in sanitized environments may be at greater Alzheimer's risk. We compare Alzheimer's rates in different countries in light of countries' historical and contemporary pathogen prevalence, sanitation, and urbanization. We find that countries that are less urbanized, with more pathogens and lower degree of sanitation have lower Alzheimer's rates.

## INTRODUCTION

Exposure to microorganisms is critical for the regulation of the immune system. The immunodysregulation of autoimmunity has been associated with insufficient microorganism exposure [1]. Global incidence patterns of autoimmune diseases reflect this aspect of their etiology: autoimmunity is inversely correlated to microbial diversity [1, 2]. The inflammation characteristic of Alzheimer’s disease (AD) shares important similarities with autoimmunity [3, 4]. The similarity in immunobiology may lead to similarity in epidemiological patterns. For this reason, here we test the hypothesis that AD incidence may be positively correlated to hygiene. The possibility that AD incidence is related to environmental sanitation was previously introduced by other authors [5, 6], and remains as of yet untested.

The ‘hygiene hypothesis’ [7] suggests that certain aspects of modern life (e.g. antibiotics, sanitation, clean drinking-water, paved roads) are associated with lower rates of exposure to microorganisms such as commensal microbiota, environmental saprophytes and helminths than would have been omnipresent during the majority of human history [8]. Low amount of microbe exposure leads to low lymphocyte turnover in the developing immune system, which can lead to immunodysregulation. Epidemiological studies have found that populations exposed to higher levels of microbial diversity exhibit lower rates of autoimmunities as well as atopies [9], a pattern that holds for countries with differing degrees of development [10, 11]. Differences in environmental sanitation can partly explain the patterns of autoimmunity and atopy across history and across world regions [2, 12]. Patient-based studies have demonstrated that individuals whose early-life circumstances were characterized by less exposure to benign infectious agents exhibit higher rates of autoimmune and atopic disorders. This pattern has been demonstrated for farm living versus rural non-farm living [13–15], daycare attendance [16, 17], more siblings [7, 18], later birth order [19–21] and pets in the household [22], exhibiting lower rates of atopic and autoimmune disorders.

Low-level persistent stimulation of the immune system leads naïve T-cells to take on a suppressive regulatory phenotype [23] necessary for regulation of both type-1 inflammation (e.g. autoimmunity) and type-2 inflammation (e.g. atopy) [12, 24, 25]. Individuals with insufficient immune stimulation may experience insufficient proliferation of regulatory T-cells (T_Regs_) [26, 27]. AD has been described as a disease of systemic inflammation [28], with the AD brain and periphery exhibiting upregulated type-1 dominant inflammation [29]: a possible sign of T_Reg_ deficiency. Immunodysregulation as a consequence of low immune stimulation may contribute to AD risk through the T-cell system. Altogether, we suggest that a hygiene hypothesis for Alzheimer’s disease (HHAD) predicts that AD incidence may be positively correlated to hygiene.

The period from gestation through childhood is typically thought to be a critical window of time during which the immune system is established [14, 30, 31], with some authors limiting this critical window to the first 2 years of life [32]. However, proliferation of T_Regs_ occurs throughout the life course: there are age-related increases in number of T_Regs_ [30, 33] with peaks at adolescence and in the sixth decade [34]. Therefore, it may be not only early-life immune stimulation that affects AD risk (and perhaps risk of other types of immunodysregulation) but also immune stimulation throughout life. Our study is designed based on the hypothesis that microorganism exposure across the lifespan may be related to AD risk.

At an epidemiological level, our prediction is opposite to Finch’s [6] hypothesis that early-life pathogen exposure should be positively correlated to AD risk. Both their and our predictions are based on speculation about T-cell differentiation, although we reached opposite suppositions. It is clear that inflammation is upregulated in AD, and Finch suggested that pathogen exposure, which is pro-inflammatory, may increase AD risk [6]. We propose that at the population level, pathogen exposure, as a proxy for benign microorganism exposure, may be protective against AD.

Griffin and Mrak [5] suggested that an HHAD is justified because hygiene-related changes in immune development are probably related to AD etiology, but it is not possible to predict the directionality of this effect. They focus on how hygiene might influence microglial activation and IL-1 expression, and the opposing effects these changes may have on AD-relevant pathways.

Our prediction, Finch’s contrary [35] prediction, and the assertion of Griffin and Mrak that further research is needed to establish the directionality of hygiene’s effect on AD motivated this empirical investigation of whether pathogen prevalence was correlated to AD rates at the country level. This information could lead to a better understanding of the environmental influences on AD etiology and could help judge the accuracy of existing predictions for future AD burden.

## METHODS

In order to test whether there is epidemiological evidence for an HHAD, data for a wide range of countries were compared. Age-standardized disability-adjusted life-year (DALY) rates (henceforth ‘AD rates’) in 2004 were evaluated in light of proxies for microbial diversity across a range of years selected to fully encompass lifespans of individuals in the AD-risk age group in 2004.

### Prevalence of Alzheimer’s and other dementias

We utilized the WHO’s Global Burden of Disease (GBD) report published in 2009, which presents data for 2004 [36]. The WHO only reports information for AD and other dementias across different countries, rather than AD alone. This variable does not include Parkinsonism [37]. While there are other types of dementia, AD accounts for between 60% and 80% of all dementia cases [38], and the neurobiological distinctions between some of the subtypes may be vague [39, 40]. The WHO report presents three variables related to AD: age-standardized DALY, age-standardized deaths, and DALY for age 60+. There is low correlation between these three measures (linear regressions after necessary data transformations had *R*-squared values 0.040; 0.089; 0.041).

The WHO’s GBD report includes the following ICD-10 codes as ‘Alzheimer’s and other dementias:’ F01, F03, G30–G31, uses an incidence-based approach, 3% time discounting, the West Level 26 and 25 life tables for all countries assuming global standard life expectancy at birth [41], standard GBD disability weights [42] and non-uniform age-weights [43, 44]. DALYs are calculated from years of lost life (YLL) and years lost due to disability (YLD). YLL data sources for AD included death registration records for 112 countries, population-based epidemiological studies, disease registers and notification systems [45]. Vital registration data with coverage over 85% were available for 76 countries, and information for the remaining 114 countries was calculated using a combination of cause-of-death modeling, regional patterns and cause-specific estimates (see [45] for details). YLD data sources for AD included disease registers, population surveys and existing epidemiological studies [43]. When only prevalence data were available, incidence statistics were modeled from estimates of prevalence, remission, fatality and background mortality using the WHO’s DisMod II software [43].

Age-standardized rates were calculated by adjusting the crude AD DALY for 5-year age groups by age-weights reflecting the age-distribution of the standard population [35]. The new WHO World Standard was developed in 2000 to best reflect projections of world age-structures for the period 2000–25 [35], and particularly closely reflects the population age-structures of low- and middle-income countries [46].

#### DALYs as better measure than death rates.

AD as cause of death is a particularly flawed measurement across different countries, as certain countries rarely attribute mortality to AD or recognize dementia as abnormal aging [47], and other causes of death may occur at far greater frequencies masking AD prevalence. The DALY measurement is the sum of years lost due to premature mortality and years spent in disability. The number of years lost due to premature mortality is based on the standard life expectancy at the age when death due to AD occurs [48]. Therefore, this measurement omits the effects of differential mortality rates previous to age 65, which accounts for the vast majority of differences in life expectancy between developed and developing countries [49], opportunely isolating the effects of later life mortality causes. Therefore, DALY includes but is not limited to AD as official cause of death, making it a more inclusive variable than AD as cause of death.

#### Age-standardized data better indicator than age 60+ data.

Age-standardized rates represent what the burden of AD would be if all countries had the same age-distribution [35]. While the WHO GBD report also provides DALY for ages 60+, these data are not age-standardized. Using DALY for ages 60+, a country with a significant population over age 85, for instance, would always appear to have greater AD incidence than a country with few people over 85, regardless of differences in age-specific AD incidence in the two countries. This is due to the exponential way in which AD risk increases with age [50, 51]. Differences in age-specific AD incidence would be masked by differences in population age structure. See Supplementary Section S6 for examples and further explanation.

### Predictive variables

The hygiene hypothesis is sometimes referred to as the ‘old friends hypothesis’ [52]. This alternative title highlights the fact that for the vast majority of our species’ history, humans would have been regularly exposed to a high degree of microbial diversity, and the human immune system was shaped through natural selection in these circumstances [53]. With rapidly increasing global urbanization beginning in the early nineteenth century, individuals began to experience diminishing exposure to these ‘friendly’ microbes due to diminishing contact with animals, feces and soil [1]. The microbes that were our ‘old friends’ previous to this epidemiological transition whose absence may lead to immunodysregulation in modern environments included gut, skin, lung and oral microbiota; orofecally transmitted bacteria, viruses and protozoa; helminthes; environmental saprophytes; and ectoparasites [1, 52]. The predictive variables in our analysis were selected for their relevance to these ‘old friends'. These variables do not specifically reflect exposure to those microbiota and commensal microorganisms that were omnipresent during our evolutionary history, but rather cover a more general collection of microbial exposures. This inclusive approach is both because of limitations of available datasets, and because it is not known if particular microbial elements specifically relate to AD etiology.

Murray and Schaller [54] assessed historical disease prevalence for the years 1944–61. Their ‘seven-item index of historical disease prevalence’ included leishmanias, schistosomes, trypanosomes, malaria, typhus, filariae and dengue. Their ‘nine-item index’ included the above diseases plus leprosy and a contemporary measure of tuberculosis. Another measure of pathogen exposure [55] combines data from the WHO and the ‘Global Infectious Diseases and Epidemiology Network’ (GIDEON) for 2002 and 2009. The WHO reports countries’ ‘percent population using improved sanitation facilities’, which ‘separate human excreta from human contact’ [56], and ‘improved drinking-water sources’, which protect the source from contamination [56]. Of 3 years for which sufficient data were available, we chose to look at 1995 (Supplementary Section S1). ‘Infant mortality rate’ (IMR) is measured as number of infant mortalities per 1000 live births [57]. We consulted historical IMR statistics [58] for years 1900–2002. The World Bank reports countries’ ‘gross national income’ (GNI) per capita at purchasing power parity (PPP) [57], the economic variable that contributes to the composite Human Development Index and is therefore an indicator of the contribution of wealth to quality of life. We looked at both the earliest year with sufficient data available, 1970 and 2004. ‘Gross domestic product’ (GDP) per capita (PPP) is an economic measure of a country divided by its mid-year population [57], often used as a measure of standard of living, although it is not a direct assessment of this. We consulted Maddison’s historical economic statistics [59] for GDP from 1900 to 2002. Children growing up in rural areas may be more exposed to pathogens due to factors including unpaved roads, contact with livestock and animal feed, and consumption of unprocessed milk [13, 14]. The World Bank reports the ‘percent of a country’s population living in urban areas’ [57], and we looked at both the earliest year with data available, 1960 and 2004.

### Statistical methods

All variables were transformed to optimize distribution symmetry to avoid undue influence by countries with extreme values for each variable (Supplementary Section S1). Linear regression was used to determine whether there was a relationship between each of the microbial exposure proxies and AD rate between countries. As there was reason to suspect predictive overlap among the variables, a principal component regression was conducted (Supplementary Section S4).

To explore which part of the lifespan has the most bearing on AD, we measure the strength of the same proxy across several years as a predictor of 2004 AD prevalence. We compare historical and contemporary disease prevalence, and GNI and urban living. Also, two of the proxies had more detailed historical information available: GDP [59] and IMR [58]. GDPs and IMRs from years spanning 1900–2002 were compared. Each year’s GDP and IMR were compared to 2004 AD rates by linear regression. The resulting regression coefficients were interpreted as the degree to which each year’s GDP or IMR and 2004 AD were correlated (Supplementary Section S5).

## RESULTS

**Figure 1.**
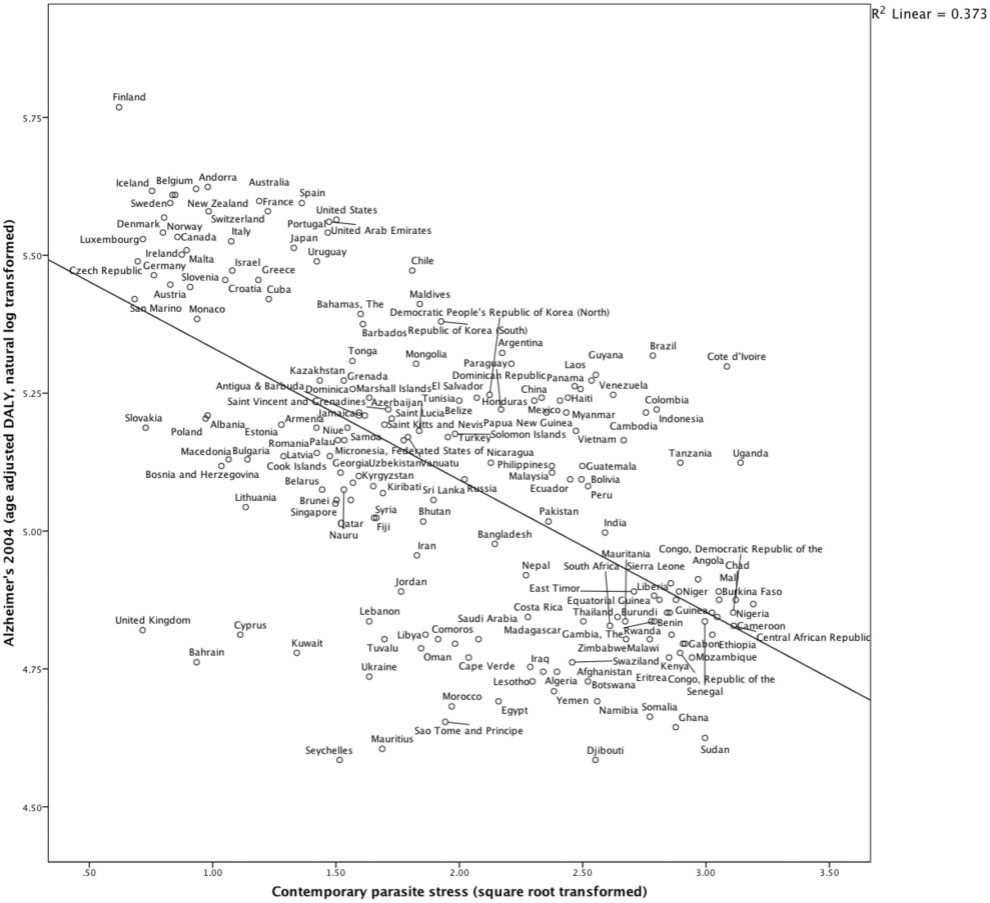
Countries’ parasite stress negatively correlated to Alzheimer's burden Countries with higher contemporary parasite prevalence have lower age-standardized rates of Alzheimer’s in 2004. *N* = 190, *R*^2 ^= 0.373, *P* < 0.0000. Contemporary parasite stress [55] combines years 2002 and 2009, and the variable is transformed by adding a constant and taking square root. The Alzheimer variable is transformed by adding a constant and taking the natural log of 2004 Alzheimer age-standardized DALY [36]

**Figure 2.**
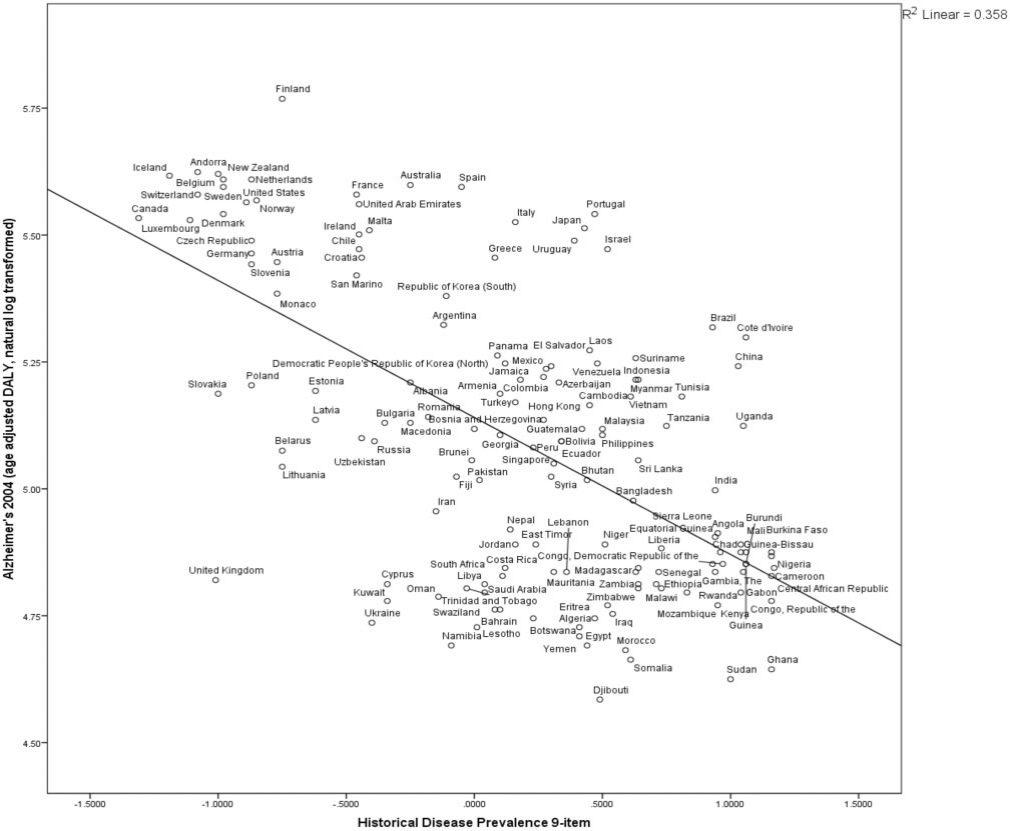
Countries’ historical disease prevalence negatively correlated to Alzheimer’s burden 2004 Countries with historically more infectious disease have lower age-standardized rates of Alzheimer’s in 2004. *N* = 148, *R*^2 ^= 0.358, *P* < 0.0000. Historical disease prevalence 9-item data compiled by Murray and Schaller for years 1944–61 [54]. Alzheimer prevalence variable is transformed by adding a constant and taking the natural log of 2004 Alzheimer age-standardized DALY [36]

**Figure 3.**
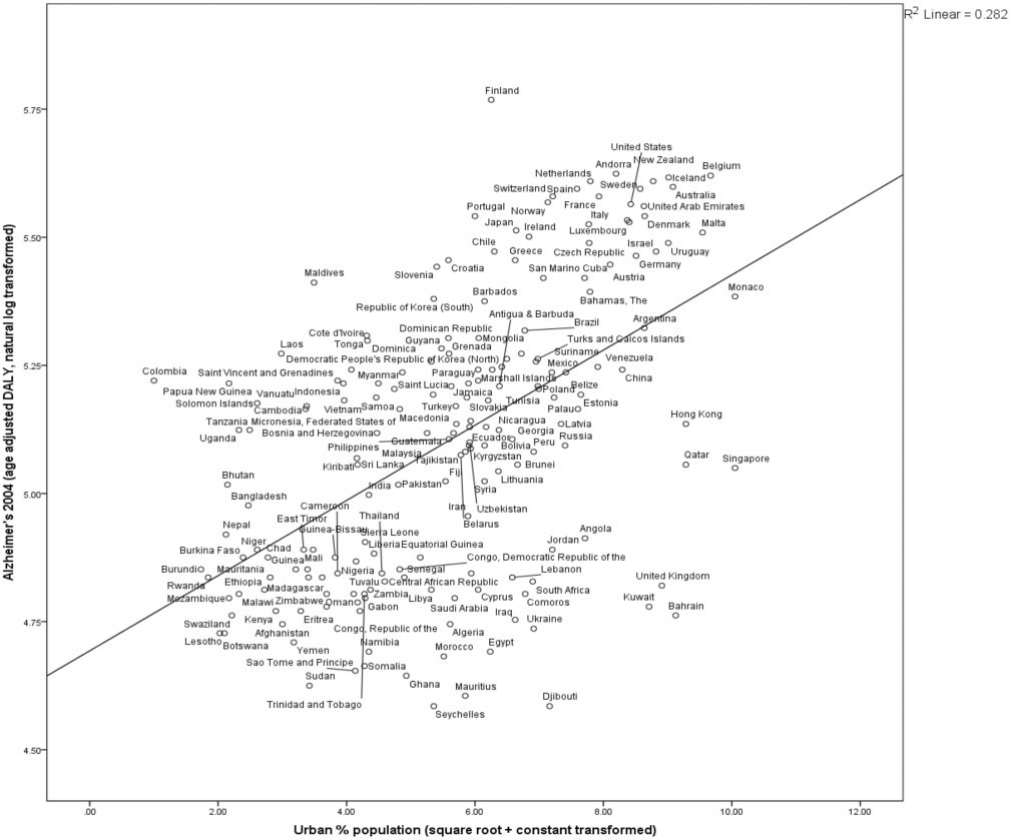
Countries’ urbanization 1960 positively correlated to Alzheimer's burden 2004 Countries with more of the population living in urban areas in 1960 have higher age-standardized rates of Alzheimer’s in 2004. *N* = 187, *R*^2 ^= 0.282, *P* < 0.0000. Urbanization data [57] are transformed by adding a constant and taking the square root. The Alzheimer prevalence variable is transformed by adding a constant and taking the natural log of 2004 Alzheimer age-standardized DALY [36]

**Figure 4.**
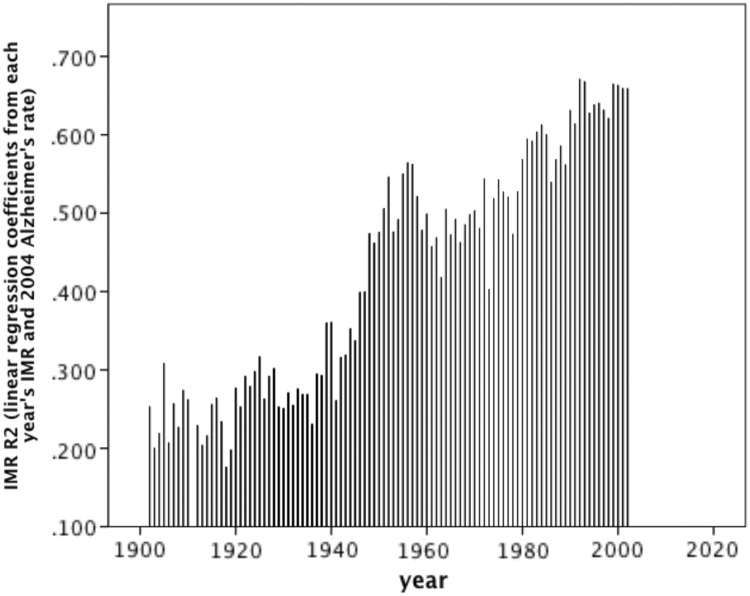
Contemporary environment may be better indicator of Alzheimer's burden than early-life environment For each year (*x*), the regression coefficient (*y*) of the correlation between year’s (*x*) IMR and AD burden in 2004. IMR for the various years were transformed by square root and natural log. See Supplementary Section S5. All correlations were significant besides the years 1900, 1901 and 1911. Significance: 1902–19, *P* < 0.05; 1920–44, *P* < 0.01; 1945–2002, *P* < 0.000

Statistical analyses revealed highly significant relationships between various measures of hygiene and age-adjusted AD DALY. High levels of pathogen exposure were associated with lower AD rates. Countries with higher disease and pathogen prevalence and IMR had lower 2004 AD rates (Table 1, Figs 1–4, Fig. S1). These results are consistent with a protective role of exposure to microbial diversity against AD, and support an HHAD.

**Table 1.** An evaluation of proxies for hygiene as predictors of Alzheimer burden

Variable	Year	*N*	*R*^2^	*P*	Direction consistent with HHAD?
Historical disease prevalence nine-item	1944–61	148	0.358	****	Yes
Historical disease prevalence seven-item	1944–61	191	0.242	****	Yes
Contemporary parasite stress	2002, 2009	190	0.373	****	Yes
Improved sanitation facilities	1995	177	0.334	****	Yes
Improved drinking-water sources	1995	178	0.327	****	Yes
GNI	1970	171	0.296	****	Yes
	2004	174	0.328	****	Yes
Urban %	1960	187	0.282	****	Yes
	2004	187	0.204	****	Yes

Linear regression for each predictive variable after transformation to optimize symmetry (Supplementary Section S1). Principal component regression for parasite stress, sanitation facilities, drinking-water sources, infant mortality, GNI and urbanization had a higher *R*^2^ than any predictive variable on its own (Supplementary Section S4). ****P < 0.0000.

Greater degree of hygiene, and therefore potentially lower degree of microorganism exposure, was associated with higher AD rates. Countries with a higher percent of the population using improved sanitation facilities, improved drinking-water sources, living in urbanized areas, higher GNI and GDP per capita (PPP) had higher rates of AD in 2004 (Table 1, Fig. 3, Figs S2–S5). The existence of a strong positive correlation between historical levels of sanitation and AD rate in 2004 is consistent with the predictions of the HHAD.

### Comparing predictive power of data from different years

Our results indicate that microbe exposure across the lifespan, not necessarily just during early-life, is associated with AD burden. Predictive variables reflecting the years during which elderly people in 2004 would have experienced their earlier years of life were not consistently better indicators of 2004 AD rate compared to years during which they would have spent their mid or later life years, consistent with observations of lifelong T_Reg_ proliferation [30, 33, 34]. Contemporary measures were more powerful indicators in the cases of contemporary parasite stress versus historical disease prevalence, and GNI in 2004 versus 1970, while percent of population living in urban areas in 1960 versus 2004 was a slightly better indicator of AD in 2004 (Fig. 3).

A series of linear regressions measuring the relationship between IMRs from 1900 to 2002 and AD in 2004 revealed that IMR was significantly negatively correlated to 2004 AD rates with an increasing annual ability to explain variance (Fig. 4, Supplementary Section S5). This analysis was restricted to countries with IMR data across 1900–2002 (*N* = 45) and thus was free from sample size biases.

Similarly, a series of linear regressions measuring the relationship between GDPs from 1900 to 2002 and AD in 2004 revealed that GDP was significantly positively correlated to 2004 AD rates, although the increasing annual ability to explain variance was only consistently true for years 1940–2002 (Fig. S7).

### Principal component analysis

A principal component regression analysis demonstrated that the combined statistical impact of the proxies for microbial diversity had a significant effect on AD rates. There were high degrees of correlation between disease prevalence, parasite stress, sanitation facilities, improved drinking water, GNI and urban population (Table S1). There was a significant relationship between the principal component and 2004 AD rate (*N* = 159, *R*^2^ = 0.425, *P* < 0.0000) (Supplementary Section S4, Fig. S6).

## DISCUSSION

Inflammation plays an important role in AD pathogenesis, and previous authors have hypothesized that immune stimulation could increase AD risk through T-cell [6] or microglial action [5, 60]. However, our results indicate that some immune stimulation may protect against AD risk. The traditional hygiene hypothesis has effectively explained prevalence rates of autoimmunity and atopy in many populations. We have found that the hygiene hypothesis can help explain some patterns in rates of AD, as predicted previously [5, 8] with a direction of correlation consistent with our predictions based on T-cell differentiation.

In support of the HHAD, we found that proxies for immune stimulation correlated to AD rates across countries. These proxies included historical disease prevalence, parasite stress, access to sanitation facilities, access to improved drinking-water sources, IMR, GNI, GDP and urbanization. These variables’ relationships to AD rate are independent of countries’ age structures.

Further evidence supporting this hypothesis from previous studies includes higher incidence of AD in developed compared to developing countries, comparison to incidence patterns of other disorders with similar immunobiology and consideration of the etiology of inflammation in AD.

### Higher Alzheimer’s risk in industrialized countries and urban environments

People living in developed compared to developing countries have higher rates of AD. AD incidence at age 80 is higher in North America and Europe than in other countries [51]. A meta-analysis found that dementia incidence doubled every 5.8 years in high income countries and every 6.7 years in low- and middle-income countries, where the overall incidence of dementia was 36% lower [39] (but see [61]). Another meta-analysis found that age-standardized AD prevalences in Latin America, China and India were all lower than in Europe, and within those regions, lower in rural compared to urban settings [62]. In a meta-analysis of Asian countries, the wealthier ones had higher age-standardized rates of AD prevalence than the poorer countries [63]. A meta-analysis of rural versus urban AD incidence demonstrated that, overall, rural living was associated with higher incidence, but when only high-quality studies were considered, rural living was associated with reduced incidence [47].

In developing countries and rural environments, there are higher rates of microbial diversity, exposure and infection. It is well-documented that rates of atopies including allergies, hay fever, eczema and asthma are lower in developing than developed countries [12, 64], as are autoimmunities such as multiple sclerosis, type-1 diabetes mellitus and Crohn’s disease [65], and the same pattern holds for rural versus urban environments [2, 14, 15] (but see [66]). We found that wealthier countries (Figs S3 and S4) and more urbanized countries (Fig. 2) had greater AD rates after adjustment for population age-structures.

### Alzheimer’s risk changes with environment

Previous studies have found that individuals from similar ethnic backgrounds living in low versus high sanitation environments exhibit low versus high risk of AD. African Americans in Indiana had higher age-specific mortality-adjusted incidence rates of AD than Yoruba in Nigeria [67], and while there is no indication that the sampled African Americans had any Yoruba or even Nigerian heritage, the results are at least consistent with our predictions for the HHAD. There is also evidence that immigrant populations exhibit AD rates intermediate between their home country and adopted country [63, 68–70]. Moving from a high-sanitation country to a low-sanitation country can decrease immigrants’ AD risk [47] [e.g. Italy to Argentina [71] (but see [72])]. Our results are consistent with the possibility that living in different countries confers different AD risk, stratified by sanitation (Figs 1 and 2).

### Alzheimer’s risk and family size

Having relatively more siblings would be expected to correspond to higher rates of early-life immune activation because of more interaction with other children who may harbor ‘friendly’ commensal microorganisms. Individuals with more siblings have lower atopy prevalence [7, 18, 20]. Certain studies have found that individuals with AD have fewer siblings than controls [73, 74], but Moceri and coworkers found no difference [75], and the opposite [76].

Younger siblings compared to first-borns would be expected to have higher rates of microorganism exposure because this would indicate higher proportion of childhood spent in contact with siblings. Later-born siblings have lower rates of atopy [7, 20, 66, 77, 78]. No birth order effect has been observed for AD [79].

### Lymphocytes and Alzheimer’s

T cells are important modulators of immune function and have been identified as the major affected system in trends attributable to the hygiene hypothesis [8]. T_Reg_ cells become more abundant with age in healthy subjects [80]. Whether the proliferation of T cells in AD [80, 81] is effector or regulatory has been the subject of controversy. Recently, Larbi *et al.* re-analyzed their earlier postulation that T_Reg_ cells may be upregulated in AD [82]. When a marker for T_Regs_, FoxP3 [83], was considered, the authors determined that the upregulation occurring was proliferation of activated effector T cells [84], consistent with our predictions for an HHAD.

One study found that T_Reg_ cells were increased in mild cognitive impairment (MCI) and T_Reg_-induced immunosuppression was stronger in MCI than in AD or controls [85]. This could be evidence that among people with predisposition toward AD (i.e. genetic or environmental risk factors), those with adequate T_Reg_ function may merely develop MCI, while those with inadequate T_Reg_ function may develop AD. Also, it may be that during AD pathogenesis, those with adequate T_Reg_ function may linger in the MCI phase for longer, while those with inadequate T_Reg_ function may progress to AD more rapidly.

It has already been well established that insufficient T_Reg_ numbers lead to excessive T_H_1 inflammation in autoimmunity and T_H_2 inflammation in atopy [52, 86]. The inflammation characteristic of AD is T_H_1 dominant [29, 87, 88]. In AD, amyloid-β activates microglia and astrocytes, which stimulate preferential T_H_1 proliferation [89], and AD patients have elevated levels of T_H_1-associated cytokines [90–92]. Non-steroidal anti-inflammatory drugs have been demonstrated to be protective against AD [93].

T cells may influence AD pathogenesis not only from within the brain but also from the periphery. Activated T_H_1 cells in the periphery could secrete pro-inflammatory cytokines, which cross the blood–brain barrier (BBB) and directly activate microglia and astrocytes in the brain, as well as indirectly induce inflammation by activating dendritic cells [89].

It should be investigated whether hygiene directly affects the development of microglia. Microglia sometimes exhibit a non-inflammatory phenotype, in contrast to the pro-inflammatory phenotype typical of the activated state in the context of AD and other brain insults [5, 94], It is unknown whether hygiene would promote development of the inflammatory or non-inflammatory phenotypes [5], and further research is needed to establish the effect of microbial deprivation on microglial development.

### ApoE

ApoE-ε4 is an allele that has pro-inflammatory effects, and increases BBB permeability [95]. Compromise of the BBB would make other pro-inflammatory mechanisms become exacerbated risk factors for AD, when otherwise, inflammation might have been limited to the periphery. There is already evidence that ApoE alleles confer different degrees of AD risk in different environments. While the ε4 allele was an AD risk factor among African Americans, this was not the case among the Yoruba in Nigeria [96], nor is ε4 associated with increased risk among Nyeri Kenyans, Tanzanians [97], Wadi Ara Arab Israelis [98], Khoi San [99], Bantu and Nilotic African cohorts [100]. These patterns support the possibility that environmental factors such as microbial diversity interact with inflammatory pathways to influence AD risk.

### Darwinian medicine

One of the major aims of Darwinian medicine is understanding human health and disease within the context of our species’ evolutionary history [101]. In their formative 1991 paper, Williams and Nesse discussed the promise for Darwinian medicine to make strides in aging research [102]. There is growing enthusiasm for Darwinian medicine approaches to understanding aging [103], specifically neurodegeneration [104] and more specifically AD (Supplementary Section S7).

The hygiene hypothesis is an important contribution from Darwinian medicine [52]. Recently, authors have suggested extending the hygiene hypothesis toward explaining obesity [105] and certain cancers [106, 107]. Thus far, the hygiene hypothesis has been discussed mostly in the context of consequences for early and mid-life pathologies, and we feel that more attention should be paid to its potential to explain patterns of disease in later life.

### Study limitations

Some critics suggest that age-weighting is not reflective of social values [42, 108]. A systematic review of GBD methodologies determined that the WHO’s report [42] represented a rigorously comprehensive methodology, but is still limited by the fact that many developing countries use paper-based health surveillance based more on estimates and projections than actual counts. It is also possible that certain predictive variables, especially urbanization, are related to surveillance accuracy.

Large-scale epidemiological studies often suffer from lack of surveillance and statistical limitations. Much of public health and epidemiological research is based on correlational studies, inherently limited by the inability to demonstrate causality. Nonetheless, these types of investigation provide necessary perspective of environmental influences on biological mechanisms, and help evaluate public health burden and predict future healthcare needs.

### Importance and applications

As AD becomes an increasingly global epidemic, there is growing need to be able to predict AD rates across world regions in order to prepare for the future public health burden [39]. It is in low- and middle-income countries that the sharpest rise is predicted to occur in the coming decades [109]. We suggest that ecological changes that will occur in low- and middle-income countries as they become more financially developed may affect AD rates in ways not currently appreciated. Other authors have also discussed the ways in which infectious disease may affect patterns of non-communicable chronic disease [110]. Disease predictions affect public policy, healthcare prioritization, research funding and resource allocation [111]. Better methods for estimating AD rates could affect these policies and strategies [112].

The next step in testing the HHAD should be to directly investigate patients’ immune activity throughout life and AD risk, preliminarily in a cross-sectional study utilizing medical records, serum analysis, or reliable interview techniques.

## SUPPLEMENTARY DATA

Supplementary data are available at *EMPH* online.

Supplementary Data

## References

[1] Rook GAW (2012). Hygiene hypothesis and autoimmune diseases. Clin Rev Allergy Immunol.

[2] Rook GAW (2007). The hygiene hypothesis and the increasing prevalence of chronic inflammatory disorders. Trans R Soc Trop Med Hyg.

[3] D’Andrea MR (2005). Add Alzheimer’s disease to the list of autoimmune diseases. Med Hypotheses.

[4] D’Andrea MR (2003). Evidence linking neuronal cell death to autoimmunity in Alzheimer’s disease. Brain Res.

[5] Griffin WST, Mrak RE, Graham AWR (2009). Is there room for Darwinian medicine and the hygiene hypothesis in Alzheimer pathogenesis?. The Hygiene Hypothesis and Darwinian Medicine.

[6] Finch CE (2010). Evolution of the human lifespan and diseases of aging: roles of infection, inflammation, and nutrition. Proc Natl Acad Sci USA.

[7] Strachan DP (1989). Hay fever, hygiene, and household size. Br Med J.

[8] Rook GAW (2009). Introduction: The changing microbial environment, Darwinian medicine and the hygiene hypothesis. The Hygiene Hypothesis and Darwinian Medicine.

[9] Lynch NR, Hagel I, Perez M (1993). Effect of anthelmintic treatment on the allergic reactivity of children in a tropical slum. J Allergy Clin Immunol.

[10] Von Mutius E, Fritzsch C, Weiland SK (1992). Prevalence of asthma and allergic disorders among children in united Germany: a descriptive comparison. Br Med J.

[11] Cookson WOCM, Moffatt MF (1997). Asthma—an epidemic in the absence of infection?. Science.

[12] Romagnani S (2004). The increased prevalence of allergy and the hygiene hypothesis: missing immune deviation, reduced immune suppression, or both?. Immunology.

[13] Leynaert B, Neukirch C, Jarvis D (2001). Does living on a farm during childhood protect against asthma, allergic rhinitis, and atopy in adulthood?. Am J Respir Crit Care Med.

[14] von Mutius E, Vercelli D (2010). Farm living: effects on childhood asthma and allergy. Nat Rev Immunol.

[15] Kilpelainen M, Terho E, Helenius H (2000). Farm environment in childhood prevents the development of allergies. Clin Exp Allergy.

[16] Haby MM, Marks GB, Peat JK (2000). Daycare attendance before the age of two protects against atopy in preschool age children. Pediatr Pulmonol.

[17] Kramer U, Heinrich J, Wjst M (1999). Age of entry to day nursery and allergy in later childhood. Lancet.

[18] Von Mutius E, Martinez FD, Fritzsch C (1994). Skin test reactivity and number of siblings. Br Med J (Clin Res Ed).

[19] Westergaard T, Rostgaard K, Wohlfahrt J (2005). Sibship characteristics and risk of allergic rhinitis and asthma. Am J Epidemiol.

[20] Matricardi PM, Franzinelli F, Franco A (1998). Sibship size, birth order, and atopy in 11,371 Italian young men. J Allergy Clin Immunol.

[21] Strachan DP, Taylor EM, Carpenter RG (1996). Family structure, neonatal infection, and hay fever in adolescence. Arch Dis Child.

[22] Apter AJ (2003). Early exposure to allergen: is this the cat’s meow, or are we barking up the wrong tree?. J Allergy Clin Immunol.

[23] Abbas AK, Lohr J, Knoechel B (2007). Balancing autoaggressive and protective T cell responses. J Autoimmun.

[24] Yazdanbakhsh M, Kremsner PG, Van Ree R (2002). Allergy, parasites, and the hygiene hypothesis. Science.

[25] Chang JS, Wiemels JL, Buffler PA (2009). Allergies and childhood leukemia. Blood Cells Mol Dis.

[26] Vukmanovic-Stejic M, Zhang Y, Cook JE (2006). Human CD4+ CD25hi Foxp3+ regulatory T cells are derived by rapid turnover of memory populations in vivo. J Clin Invest.

[27] Sakaguchi S, Miyara M, Costantino CM (2010). FOXP3+ regulatory T cells in the human immune system. Nat Rev Immunol.

[28] Pellicano M, Larbi A, Goldeck D (2011). Immune profiling of Alzheimer patients. J Neuroimmunol.

[29] Schwarz MJ, Chiang S, Muller N (2001). T-helper-1 and T-helper-2 responses in psychiatric disorders. Brain Behav Immun.

[30] Prescott SL (2008). Promoting tolerance in early life: pathways and pitfalls. Curr Allergy Clin Immunol.

[31] Smith M, Tourigny MR, Noakes P (2008). Children with egg allergy have evidence of reduced neonatal CD4+ CD25+ CD127 lo/- regulatory T cell function. J Allergy Clin Immunol.

[32] Nesse RM, Williams GC Allergy. Why We Get Sick: The New Science of Darwinian Medicine.

[33] Gregg R, Smith C, Clark F (2005). The number of human peripheral blood CD4+ CD25high regulatory T cells increases with age. Clin Exp Immunol.

[34] Caetano Faria AM, Monteiro de Moraes S, Ferreira de Freitas LH (2008). Variation rhythms of lymphocyte subsets during healthy aging. Neuroimmunomodulation.

[35] Ahmad OB, Boschi-Pinto C, Lopez AD (2001). Age Standardization of Rates: A New WHO Standard.

[36] World Health Organization Burden of Disease. Health Statistics and Health Information Systems: Disease and Injury Country Estimates.

[37] World Health Organization Deaths and DALYs 2004: Analysis Categories and Mortality Data Sources (Annex C). Global Burden of Disease: 2004 Update.

[38] Alzheimer’s Association (2012). 2012 Alzheimer’s disease facts and figures. Alzheimer Dement.

[39] Sosa-Ortiz AL, Acosta-Castillo I, Prince MJ (2012). Epidemiology of dementias and Alzheimer’s disease. Arch Med Res.

[40] Ince P (2001). Pathological correlates of late-onset dementia in a multicentre, community-based population in England and Wales. Lancet.

[41] Mathers C, Vos T, Lopez A (2001). National Burden of Disease Studies: A Practical Guide. WHO Global Program on Evidence for Health Policy.

[42] Polinder S, Haagsma JA, Stein C (2012). Systematic review of general burden of disease studies using disability-adjusted life years. Popul Health Metr.

[43] Mathers CD, Lopez AD, Murray CJ, Lopez AD, Mathers CD, Ezzati M, Jamison DT (2006). The burden of disease and mortality by condition: data, methods, and results for 2001. Global Burden of Disease and Risk Factors.

[44] Williams A (1999). Calculating the global burden of disease: time for a strategic reappraisal?. Health Econ.

[45] Mathers C, Fat DM, Boerma J (2008). The Global Burden of Disease: 2004 Update.

[46] World Health Organization Information on Estimation Methods. Global Health Observatory (GHO).

[47] Russ TC, Batty GD, Hearnshaw GF (2012). Geographical variation in dementia: systematic review with meta-analysis. Int J Epidemiol.

[48] World Health Organization Mortality and Burden of Disease Estimates for WHO Member States in 2004.

[49] Kalaria RN, Maestre GE, Arizaga R (2008). Alzheimer’s disease and vascular dementia in developing countries: prevalence, management, and risk factors. Lancet Neurol.

[50] Brookmeyer R, Gray S, Kawas C (1998). Projections of Alzheimer’s disease in the United States and the public health impact of delaying disease onset. Am J Public Health.

[51] Ziegler-Graham K, Brookmeyer R, Johnson E (2008). Worldwide variation in the doubling time of Alzheimer’s disease incidence rates. Alzheimers Dement.

[52] Rook G (2010). 99th Dahlem conference on infection, inflammation and chronic inflammatory disorders: Darwinian medicine and the “hygiene” or “old friends” hypothesis. Clin Exp Immunol.

[53] Nesse RM, Williams GC (1996). Why We Get Sick: The New Science of Darwinian Medicine.

[54] Murray DR, Schaller M (2010). Historical prevalence of infectious diseases within 230 geopolitical regions: A tool for investigating origins of culture. J Cross Cult Psychol.

[55] Fincher CL, Thornhill R (2012). Parasite-stress promotes in-group assortative sociality: the cases of strong family ties and heightened religiosity. Behav Brain Sci.

[56] World Health Organization MDG 7: Environment Sustainability. Global Health Observatory Data Repository.

[57] The World Bank World Development Indicators.

[58] Abouharb MR, Kimball AL (2007). A new dataset on infant mortality rates, 1816–2002. J Peace Res.

[59] Maddison A (2003). The World Economy: Historical Statistics.

[60] Stanley LC, Mrak RE, Woody RC (1994). Glial cytokines as neuropathogenic factors in HIV infection: pathogenic similarities to Alzheimer’s disease. J Neuropathol Exp Neurol.

[61] World Health Organization (2012). Dementia: A Public Health Priority.

[62] Rodriguez JJL, Ferri CP, Acosta D (2008). Prevalence of dementia in Latin America, India, and China: a population-based cross-sectional survey. Lancet.

[63] Venketasubramanian N, Sahadevan S, Kua E (2010). Interethnic differences in dementia epidemiology: global and Asia-Pacific perspectives. Dement Geriatr Cogn Disord.

[64] Beasley R (1998). Worldwide variation in prevalence of symptoms of asthma, allergic rhinoconjunctivitis, and atopic eczema: ISAAC. Lancet.

[65] Bach JF (2002). The effect of infections on susceptibility to autoimmune and allergic diseases. New Engl J Med.

[66] Tallman PS, Kuzawa C, Adair L (2012). Microbial exposures in infancy predict levels of the immunoregulatory cytokine interleukin-4 in filipino young adults. Am J Hum Biol.

[67] Hendrie HC, Osuntokun BO, Hall KS (1995). Prevalence of Alzheimer’s disease and dementia in two communities: Nigerian Africans and African Americans. Am J Psychiatry.

[68] Yamada T, Kadekaru H, Matsumoto S (2002). Prevalence of dementia in the older Japanese-Brazilian population. Psychiatry Clin Neurosci.

[69] Graves A, Larson E, Edland S (1996). Prevalence of dementia and its subtypes in the Japanese American population of King County, Washington State The Kame Project. Am J Epidemiol.

[70] Hendrie HC, Ogunniyi A, Hall KS (2001). Incidence of dementia and Alzheimer disease in 2 communities. JAMA.

[71] Roman G (1998). Gene-environment interactions in the Italy-Argentina “Colombo 2000” project. Funct Neurol.

[72] Adelman S, Blanchard M, Rait G (2011). Prevalence of dementia in African-Caribbean compared with UK-born White older people: two-stage cross-sectional study. Br J Psychiatry.

[73] Kim J, Stewart R, Shin I (2003). Limb length and dementia in an older Korean population. J Neurol Neurosurg Psychiatry.

[74] Alafuzoff I, Almqvist E, Adolfsson R (1994). A comparison of multiplex and simplex families with Alzheimer’s disease/senile dementia of Alzheimer type within a well defined population. J Neural Transm Park Dis Dement Sect.

[75] Moceri VM, Kukull WA, Emanual I (2001). Using census data and birth certificates to reconstruct the early-life socioeconomic environment and the relation to the development of Alzheimer’s disease. Epidemiology.

[76] Moceri VM, Kukull W, Emanuel I (2000). Early-life risk factors and the development of Alzheimer’s disease. Neurology.

[77] Strachan DP, Harkins LS, Johnston IDA (1997). Childhood antecedents of allergic sensitization in young British adults. J Allergy Clin Immunol.

[78] Jarvis D, Chinn S, Luczynska C (1997). The association of family size with atopy and atopic disease. Clin Exp Allergy.

[79] Knesevich JW, LaBarge E, Martin RL (1982). Birth order and maternal age effect in dementia of the Alzheimer type. Psychiatry Res.

[80] Rosenkranz D, Weyer S, Tolosa E (2007). Higher frequency of regulatory T cells in the elderly and increased suppressive activity in neurodegeneration. J Neuroimmunol.

[81] Togo T, Akiyama H, Iseki E (2002). Occurrence of T cells in the brain of Alzheimer’s disease and other neurological diseases. J Neuroimmunol.

[82] Larbi A, Pawelec G, Witkowski JM (2009). Dramatic shifts in circulating CD4 but not CD8 T cell subsets in mild Alzheimer’s disease. J Alzheimers Dis.

[83] Vaeth M, Schliesser U, Muller G (2013). Dependence on nuclear factor of activated T-cells (NFAT) levels discriminates conventional T cells from Foxp3+ regulatory T cells. Proc Natl Acad Sci.

[84] Pellicano M, Larbi A, Goldeck D (2012). Immune profiling of Alzheimer patients. J Neuroimmunol.

[85] Saresella M, Calabrese E, Marventano I (2010). PD1 negative and PD1 positive CD4+ T regulatory cells in mild cognitive impairment and Alzheimer’s disease. J Alzheimers Dis.

[86] Poojary KV, Yi-chi MK, Farrar MA (2010). Control of Th2-mediated inflammation by regulatory T cells. Am J Pathol.

[87] Remarque E, Bollen E, Weverling-Rijnsburger A (2001). Patients with Alzheimer’s disease display a pro-inflammatory phenotype. Exp Gerontol.

[88] Singh V, Mehrotra S, Agarwal S (1999). The paradigm of Th1 and Th2 cytokines. Immunol Res.

[89] Town T, Tan J, Flavell RA (2005). T-cells in Alzheimer’s disease. Neuromolecular Med.

[90] Heneka MT, O’Banion MK (2007). Inflammatory processes in Alzheimer’s disease. J Neuroimmunol.

[91] Swardfager W, Lanctot K, Rothenburg L (2010). A meta-analysis of cytokines in Alzheimer’s disease. Biol Psychiatry.

[92] Huberman M, Shalit F, Roth-Deri I (1994). Correlation of cytokine secretion by mononuclear cells of Alzheimer patients and their disease stage. J Neuroimmunol.

[93] McGeer PL, Schulzer M, McGeer EG (1996). Arthritis and anti-inflammatory agents as possible protective factors for Alzheimer’s disease. Neurology.

[94] Morgan D (2006). Modulation of microglial activation state following passive immunization in amyloid depositing transgenic mice. Neurochem Int.

[95] Donahue JE, Johanson CE (2008). Apolipoprotein E, amyloid-[beta], and blood-brain barrier permeability in Alzheimer disease. J Neuropathol Exp Neurol.

[96] Osuntokun BO, Sahota A, Ogunniyi A (2004). Lack of an association between apolipoprotein E e4 and Alzheimer’s disease in elderly Nigerians. Ann Neurol.

[97] Sayi J, Patel N, Premukumar D (1997). Apolipoprotein E polymorphism in elderly east Africans. East Afr Med J.

[98] Farrer LA, Friedland RP, Bowirrat A (2003). Genetic and environmental epidemiology of Alzheimer’s disease in Arabs residing in Israel. J Mol Neurosci.

[99] Sandholzer C, Delport R, Vermaak H (1995). High frequency of the apo e4 allele in Khoi San from South Africa. Hum Genet.

[100] Chen CH, Mizuno T, Elston R (2010). A comparative study to screen dementia and APOE genotypes in an ageing East African population. Neurobiol Aging.

[101] Nesse RM (2001). How is Darwinian medicine useful?. West J Med.

[102] Williams GC, Nesse RM (1991). The dawn of Darwinian medicine. Q Rev Biol.

[103] Williams G (1957). Pleiotropy, natural selection, and the evolution of senescence. Evolution.

[104] Frank SA (2010). Somatic evolutionary genomics: mutations during development cause highly variable genetic mosaicism with risk of cancer and neurodegeneration. Proc Natl Acad Sci.

[105] Isolauri E, Kalliomaki M, Rautava S (2010). Obesity—extending the hygiene hypothesis. Microbial–Host Interaction: Tolerance versus Allergy, Vol. 64. Nestlé Nutrition Institute Workshop Series: Pediatric Program.

[106] Erdman SE, Rao VP, Olipitz W (2010). Unifying roles for regulatory T cells and inflammation in cancer. Int J Cancer.

[107] Erdman SE, Poutahidis T (2010). Roles for inflammation and regulatory T cells in colon cancer. Toxicol Pathol.

[108] Anand S, Hanson K (1997). Disability-adjusted life years: a critical review. J Health Econ.

[109] Prince M, Jackson J (2009). World Alzheimer’s Report Executive Summary. Alzheimer’s Disease International.

[110] Remais JV, Zeng G, Li G (2013). Convergence of non-communicable and infectious diseases in low- and middle-income countries. Int J Epidemiol.

[111] Lopez AD, Mathers CD, Ezzati M, Lopez AD, Mathers CD, Ezzati M (2006). Measuring the global burden of disease and risk factors, 1990–2001. Global Burden of Disease and Risk Factors.

[112] Bloom BS, de Pouvourville N, Straus WL (2003). Cost of illness of Alzheimer’s disease: how useful are current estimates?. Gerontologist.

