# Supplementary material for: Hygiene and the world distribution of Alzheimer’s disease: Epidemiological evidence for a relationship between microbial environment and age-adjusted disease burden
Source: Evol Med Public Health. 2013 Jul 11;2013(1):173–86. doi: 10.1093/emph/eot015 (PMC3868447; doi:10.1093/emph/eot015)
Supplement: Supplementary Data [file supp_2013_1_173__index.html]

Hygiene and the world distribution of Alzheimer's Disease — Hygiene and the world distribution of Alzheimer’s disease — Supplementary Data 

# Hygiene and the world distribution of Alzheimer’s disease

## Supplementary Data

files

**Files in this Data Supplement:**

- Supplementary Data - docx file
