# Supplementary material for: Hygiene and the world distribution of Alzheimer’s disease: Epidemiological evidence for a relationship between microbial environment and age-adjusted disease burden
Source: Evol Med Public Health. 2013 Jul 11;2013(1):173–86. doi: 10.1093/emph/eot015 (PMC3868447; doi:10.1093/emph/eot015)
Supplement: Supplementary Data [file supp_eot015_FoxSupplement.docx]

Supplementary Material

**Title**: Hygiene and the world distribution of Alzheimer’s Disease

Table of Contents

1. Transforming data: 2

2. Results: 3

3. Predictive variable correlations: 8

4. Principal component analysis: 9

5. Series of linear regressions: 11

6. Age-standardized rather than 60+ DALY: 12

7. Supplemental references: 14

# 1. Transforming data:

In order to render the data appropriate for regression analysis, variables were transformed to maximize symmetry. Those variables that did not meet the criteria for normal distribution were transformed using natural logarithm or square root transformations with constants added and inverses taken when necessary.

1. Alzheimer’s Disease-altered life years, age-adjusted:

Age-standardized DALYs per 100,000, 2004

Ln (DALYAA + 1)

2. Historical disease prevalence 9-item: This variable was already normally distributed, and no transformation improved distribution.

3. Historical disease prevalence 7-item: This variable was already normally distributed, and no transformation improved distribution.

4. Improved sanitation facilities 1995:

SanFacT=sqrt(101 - SanFac).

SanFacTreflect=7 - SanFacT.

Data with sufficient sample size was available for 1995, 2000, and 2005. The latter was ruled out for this analysis as our dependent variable, Alzheimer incidence, was for an earlier year. We chose to look at 1995 rather than 2000 because 1995 would better capture environmental circumstances before 2004 AD patients experienced onset.

5. Improved drinking-water sources 1995:

ImpWt=sqrt(101 - ImpW).

ImpWtinverse=11 - ImpWt.

6. GNI 1970

lnGNI=ln(GNI70).

7. GNI 2004

lnGNI=ln(GNI04).

8. Urban population 1960:

sqrtUrban = sqrt(Urban + 1)

9. Urban population 2004: No transformation necessary

10. Percent over population over age 65:

sqrt(Per65)

11. Sqrt(Combined parasite-stress + 4)

# 2. Results:


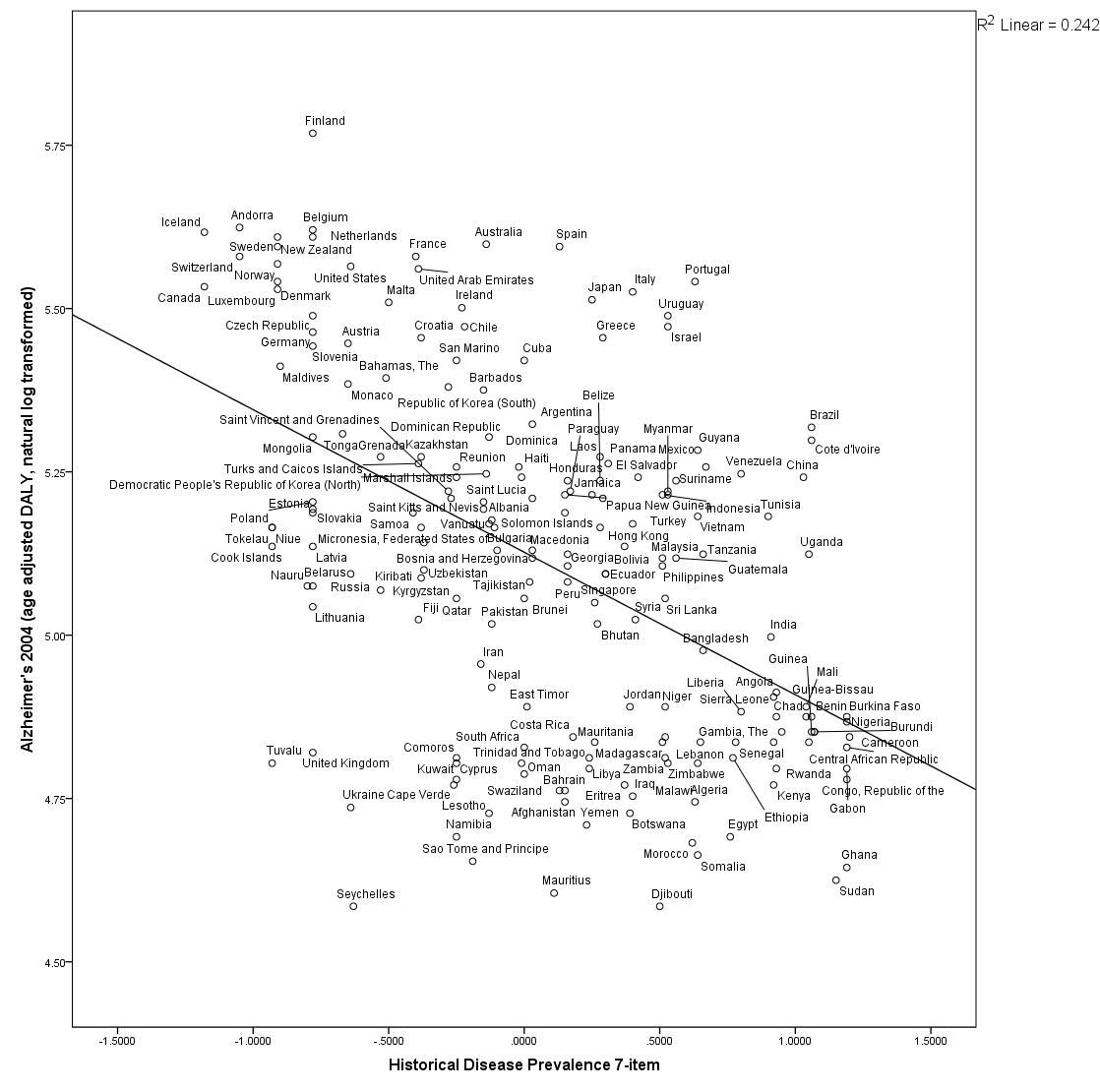


Figure S 1. Countries’ historical disease prevalence negatively correlated with AD burden 2004

**Caption**: Countries with historically more infectious disease have lower age-standardized rates of Alzheimer’s in 2004. N=191, R^2^=0.242, p<0.0000. Historical disease prevalence 7-item data compiled by Murray and Schaller for years (1944-1961) (Murray & Schaller 2010). Alzheimer burden here is the natural log of Alzheimer age-standardized disease-adjusted life-years 2004 as reported in the WHO Burden of Disease report published 2009 (World_Health_Organization 2009).


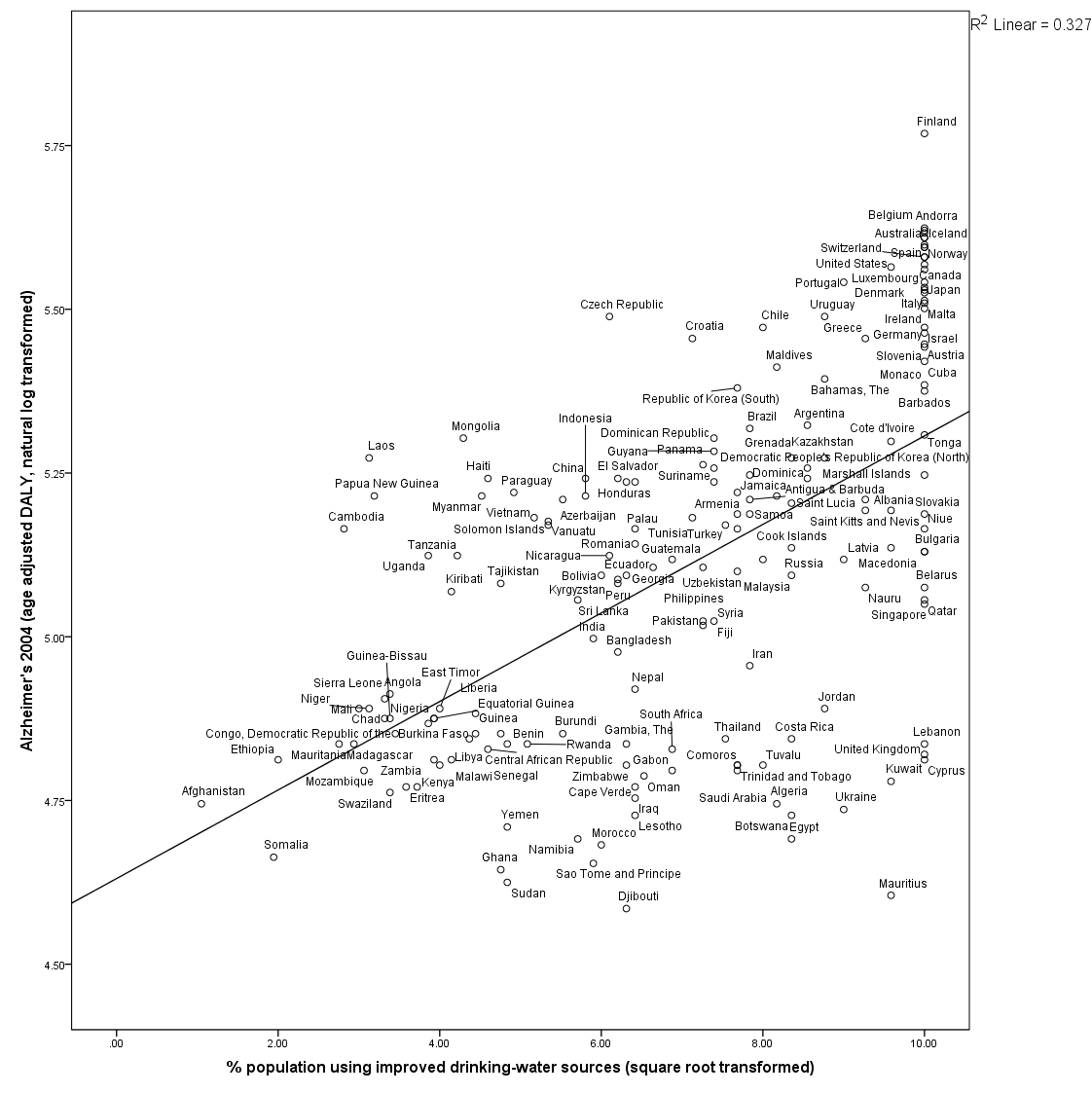


Figure S 2. Countries’ use of improved drinking-water sources 1995 positively correlated with AD burden 2004

**Caption**: Countries with historically more of population using improved drinking-water sources have higher age-standardized rates of Alzheimer’s in 2004. N=178, R^2^=0.327, p<0.0000. Sanitation data from WHO (World_Health_Organization 2011). Alzheimer burden here is the natural log of Alzheimer age-standardized disease-adjusted life-years 2004 as reported in the WHO Burden of Disease report published 2009 (World_Health_Organization 2009).


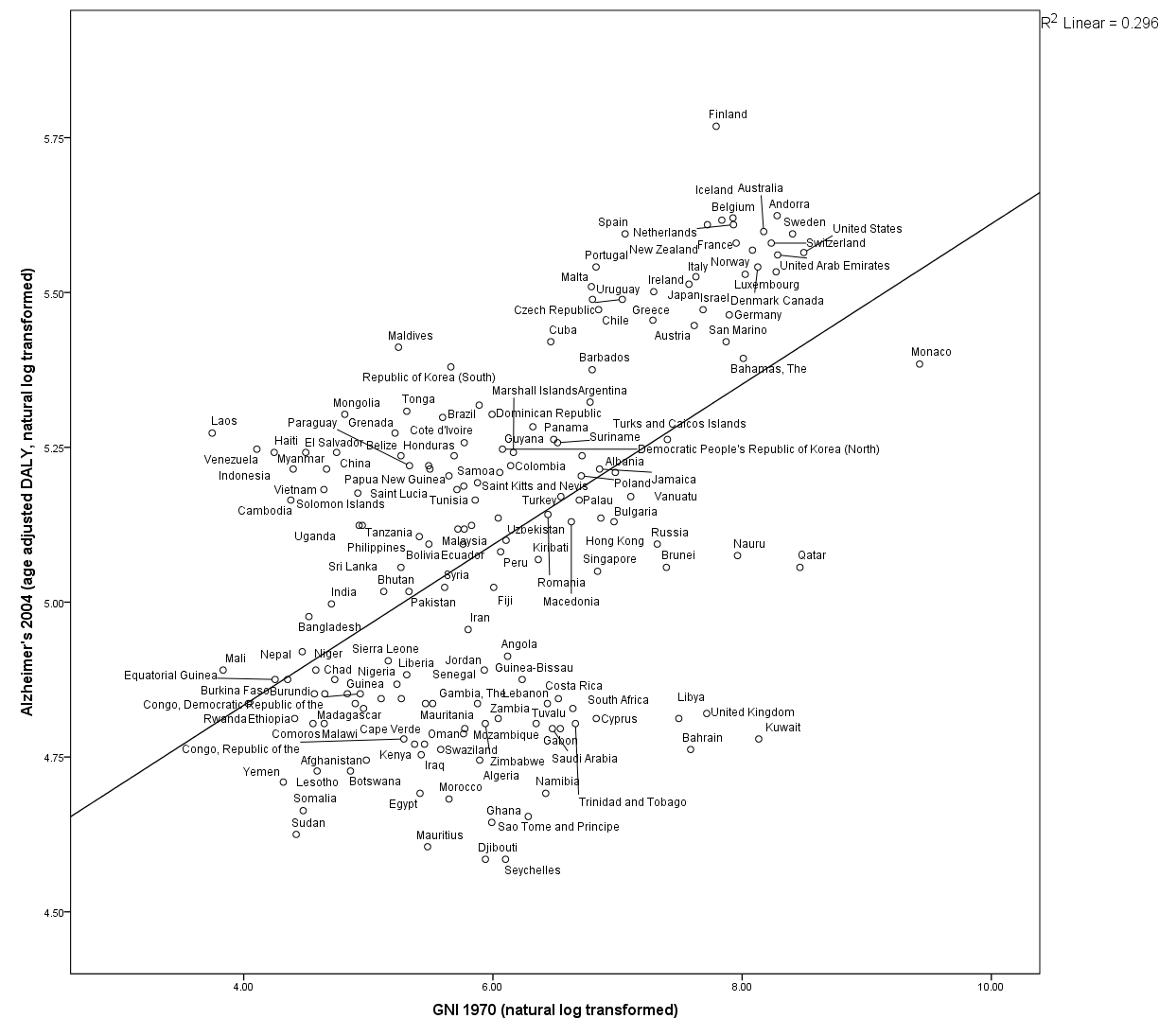


Figure S 3. Countries’ GNI 1970 positively correlated with AD burden 2004

**Caption**: Countries with higher GNI have higher age-standardized rates of Alzheimer’s in 2004. N=171, R^2^=0.296, p<0.0000. GNI data comes from World Bank (The_World_Bank 2011). Alzheimer burden here is the natural log of Alzheimer age-standardized disease-adjusted life-years 2004 as reported in the WHO Burden of Disease report published 2009 (World_Health_Organization 2009).


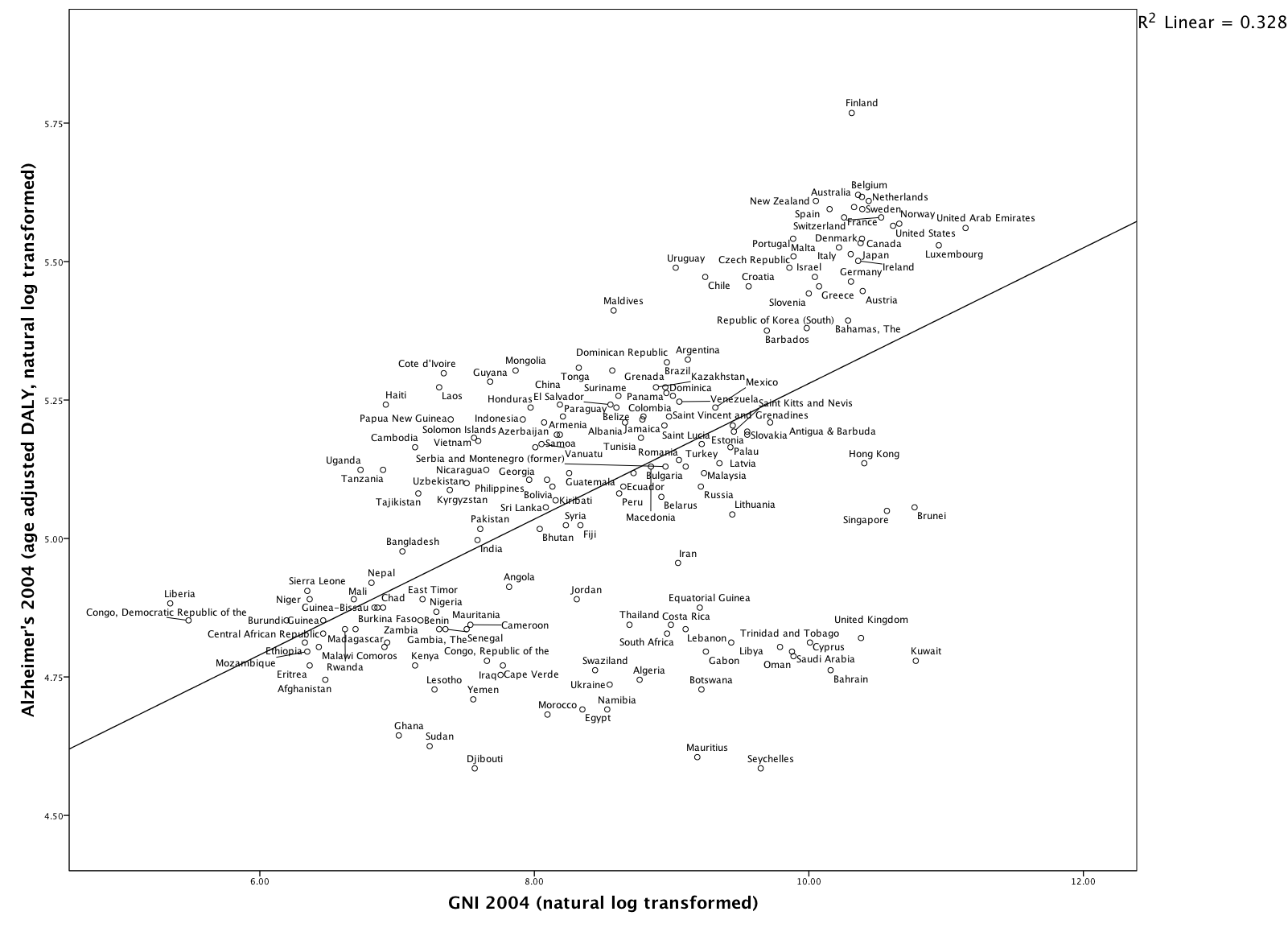


**Figure S4**: Wealthier countries have higher age-standardized rates of Alzheimer’s in 2004.

**Caption:** N=174, R^2^=0.328, p<0.0000. GNI (The_World_Bank 2011) is natural log transformed. The Alzheimer variable is transformed by adding a constant and taking the natural log of 2004 Alzheimer age-standardized DALY (World_Health_Organization 2009).


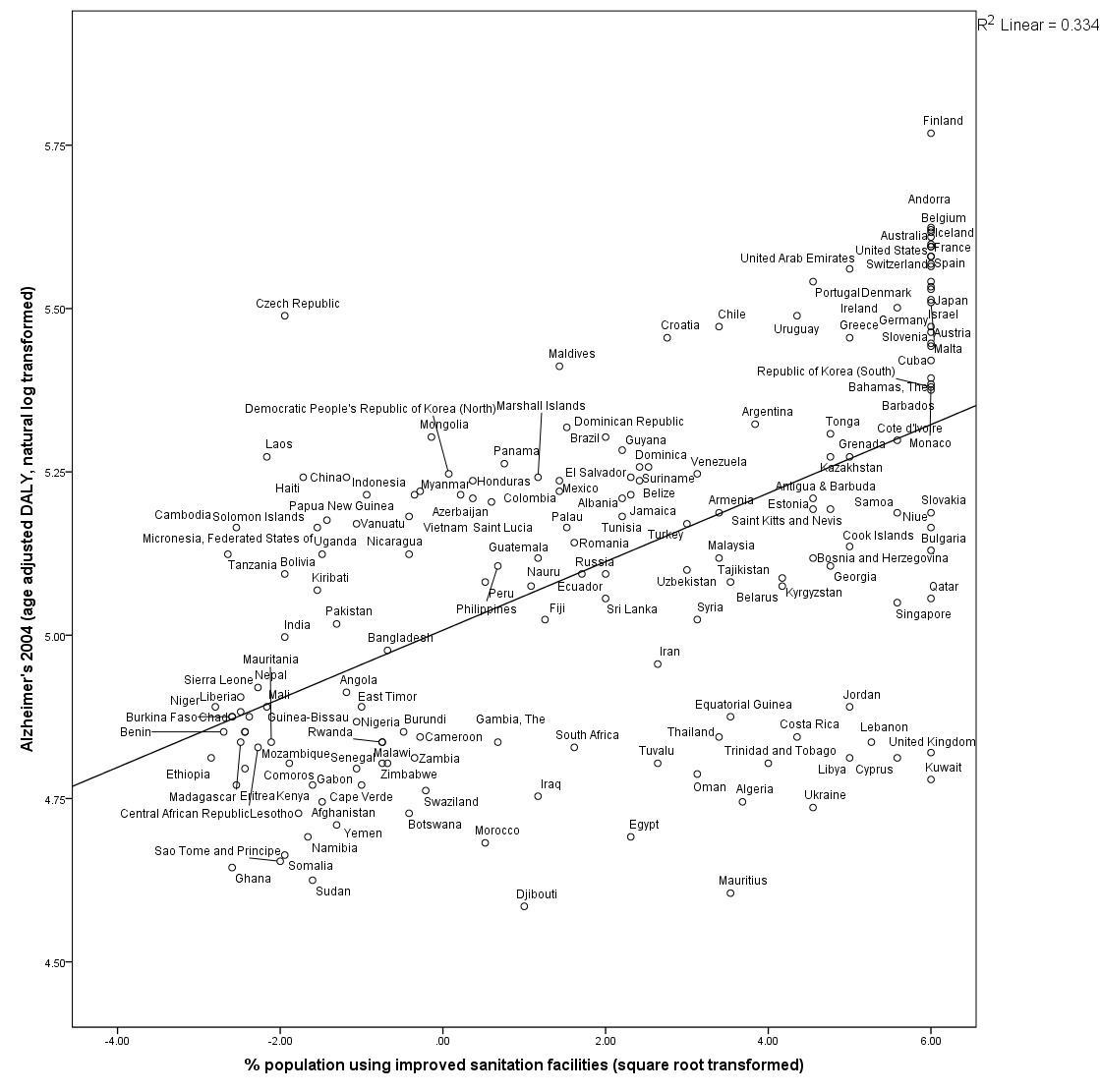


Figure S5. Countries’ use of sanitation facilities 1995 positively correlated with AD burden 2004

**Caption**: Countries with historically more of population using improved sanitation facilities have higher age-standardized rates of Alzheimer’s in 2004. N=177, R^2^=0.334, p<0.0000. Sanitation data from WHO (73) power transformed. The Alzheimer variable is transformed by adding a constant and taking the natural log of 2004 Alzheimer age-standardized DALY (65).

# 3. Predictive variable correlations:


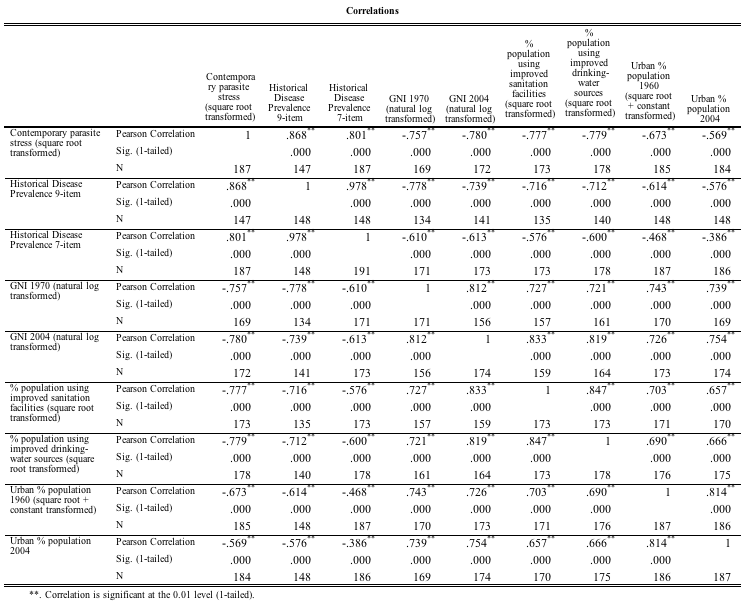


** Correlation is significant at the 0.01 level (1-tailed).

Table S 1. Correlations between predictive variables in analysis

The proxies for hygiene were highly correlated with one another.

# 4. Principal component analysis:

A principal component was computed to calculate the cumulative effect of proxies for immunoactivation on 2004 AD DALY age-adjusted. Of the repeated proxies (e.g. GNI 1970 and 2004), the one with higher R2 value was used (e.g. GNI 1970).

| Component | Initial Eigenvalues | | |
| --- | --- | --- | --- |
|  | Total | % of Variance | Cumulative % |
| 1 | 4.039 | 80.779 | 80.779 |
| 2 | .382 | 7.633 | 88.412 |
| 3 | .260 | 5.196 | 93.608 |
| 4 | .179 | 3.571 | 97.179 |
| 5 | .141 | 2.821 | 100.000 |

Table S 2. Principal component: Eigenvalues

To create the component, the resulting variable was multiplied by the square root of 4.039, the Eigenvalue.


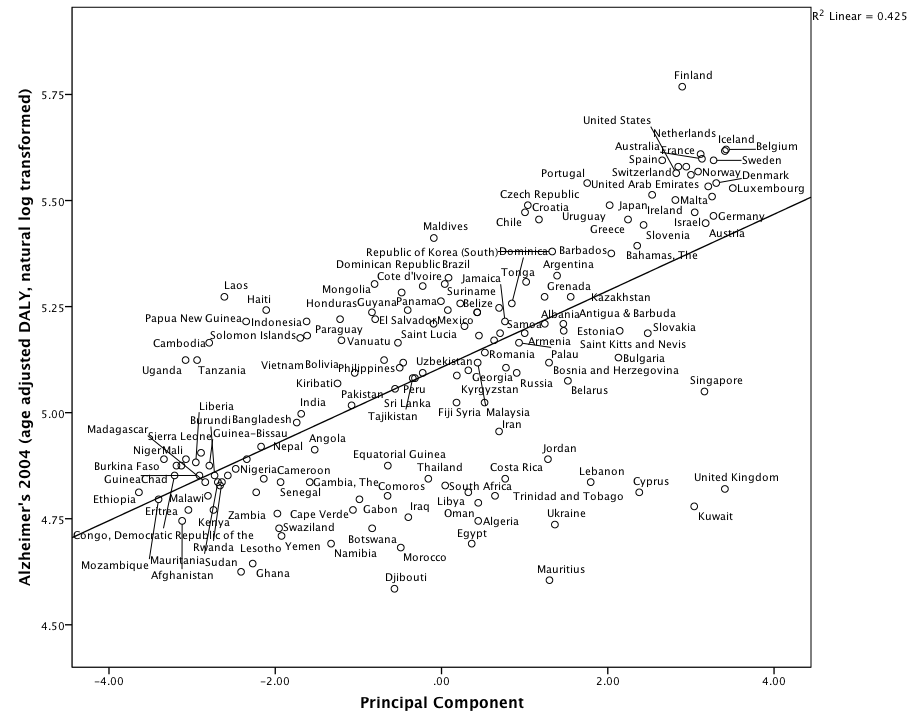


Figure S 6. Linear regression using principal component and Alzheimer burden 2004 (transformed)

The principal component was highly correlated with AD burden in 2004

N = 159

R^2^ = 0.425061

p < 0.00000

# 5. Series of linear regressions:

Using Maddison’s historical economic data (see (Maddison 2003)) and IMR data as compiled by Abouharb and Kimball (Abouharb & Kimball 2007), variables for each year’s GDP and IMR were transformed to optimize symmetry, either by square root or natural log transformation, based on histogram and Kolmogorov-Smirnov p value less than 0.05. This analysis only included those cases with data for years 1900-2002. 2002 was selected as the cutoff point because IMR data only went to this date from this particular source. Next, I performed 102 linear regressions for each variable. Each test was for a different year, for instance, the first test looked at the relationship between 1900 GDP and 2004 AD rates.

Linear regressions between each year’s GDP (square root or log transformed) and Alzheimer prevalence 2004. Data not listed but plotted in Figure S7. Significance was as follows: 1900-1912 and 1914-1919 p < 0.01, 1913 and 1920-2002 p < 0.000. Two cases (i.e. countries) were excluded as outliers to avoid undue influence on the model fit.

Linear regressions between each year’s IMR (square root or log transformed) and Alzheimer prevalence 2004. Data not listed but plotted in Figure 3 of manuscript.

N/A: not applicable

NS: not significant

Transformations:

1900-1940: LN

1941-1954: SQRT

1955-2002: LN

All correlations were highly significant besides the years 1900, 1901, and 1911. Significance was as follows: 1902-1919 p < 0.05, 1920-1944 p < 0.01, 1945-2002 p < 0.000. One case (i.e. country) was excluded as an outlier to avoid undue influence on the model fit.


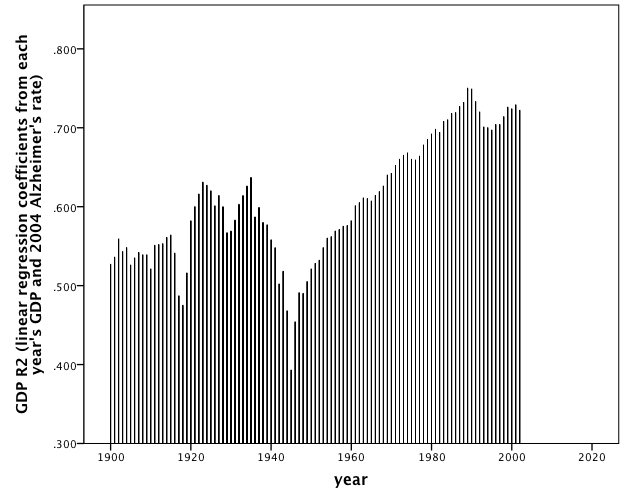


Figure S 7. How well does GDP for years 1900-2002 predict 2004 AD rate?

For each year (x), the regression coefficient (y) of the correlation between year’s (x) GDP and AD rate in 2004. IMR for the various years were transformed by square root or natural log. This analysis was restricted to countries with IMR data across 1900-2002 (N = 46) thus was free from sample size biases, and two cases were excluded as outliers based on Mahalanobis and Cook’s distances to avoid undue influence over the model fit. All correlations highly significant (p < 0.00000).

# 6. Age-standardized rather than 60+ DALY:

Mongolia and Germany (Table S3) have the same historical disease prevalence, which is in the 15th percentile of all countries, but Mongolia’s percent of population age 65+ is low (35th percentile) and Germany’s is high (98th percentile). These percentiles exactly match these countries’ AD DALY for ages 60+ percentiles, indicating that AD DALY 60+ may be unduly influenced by percent of population age 60+. On the other hand, age-standardized AD DALY rates in Mongolia and Germany are similar to one another, mirroring the similarity between their historical disease prevalences rather than the vast difference between their percentages of population over age 65 (Table S3). Age-standardized AD DALY is a more accurate measurement of individuals’ AD risk than AD DALY for age 60+, a measurement too heavily influenced by a population’s age distribution to be informative about risk factors for AD besides age.

|  |  | Mongolia | Germany |
| --- | --- | --- | --- |
| Percent of population over 65 | Value | 3.92 | 19.07 |
|  | Percentile | 35 | 98 |
| Historical Disease Prevalence 7-item | Value | -0.78 | -0.78 |
|  | Percentile | 15 | 15 |
| Alzheimer DALY over age 60 | Value | 2 | 375 |
|  | Percentile | 35 | 98 |
| Alzheimer DALY age-standardized | Value | 200 | 235 |
|  | Percentile | 78 | 86 |

Table S 3. Choosing an appropriate measure of Alzheimer prevalence

**Caption**: The WHO Burden of Disease report presents three different ways to measure Alzheimer prevalence. Our analysis aims to gauge Alzheimer risk for individuals within each country based on degree of sanitation within that country during their earliest years of life, and thus we are not concerned with life expectancy or population age structure. Here, we consider two countries with similar historical pathogen prevalence but different population age structures, and compare the measures of Alzheimer risk for each. It appears that age-standardized Alzheimer DALY better isolates the effects of risk factors other than population age structure, compared with DALY for age 60+.

### Population age-structures

Currently, epidemiological research largely operates on the premise that AD risk is primarily a function of age. Based on this idea, differences in age structure and age-specific mortality rates between countries would essentially account for differences in AD rates between countries. This conventional wisdom confuses individual-specific AD risk with number of people with AD, the latter figure determined heavily by proportion of older individuals in populations.

# 7. Supplemental references:

**Extra references for manuscript:**

**Existing evolutionary hypotheses for AD:**

Glass DJ & Arnold SE (2011) Some evolutionary perspectives on Alzheimer's disease pathogenesis and pathology. Alzheimer's and Dementia in press:1-9.

Sapolsky RM & Finch CE (2000) Alzheimer's disease and some speculations about the evolution of its modifiers. Ann. N. Y. Acad. Sci. 924(1):99-103.

Finch CE & Sapolsky RM (1999) The evolution of Alzheimer disease, the reproductive schedule, and apoE isoforms. Neurobiol. Aging 20(4):407-428.

Finch CE & Stanford CB (2004) Meat-adaptive genes and the evolution of slower aging in humans. The Quarterly review of biology 79(1):3-50.

Finch CE & Morgan TE (2007) Systemic inflammation, infection, ApoE alleles, and Alzheimer disease: a position paper. Curr. Alzheimer Res. 4(2):185-189.

Reser J (2009) Alzheimer's disease and natural cognitive aging may represent adaptive metabolism reduction programs. Behav. Brain Funct. 5(1):13-27.

Bufill E & Blesa R (2006) Alzheimer's disease and brain evolution: is Alzheimer's disease an example of antagonistic pleiotropy? Rev. Neurol. 42(1):25-33.

Rapoport SI (1989) Hypothesis: Alzheimer's Disease is a Phylogenetic Disease. Med. Hypotheses 29:147-150.

Powell A. Alzheimer's for humans only. Harvard Gazette.

**Supplementary Material references:**

Abouharb MR, Kimball AL: A New Dataset on Infant Mortality Rates, 1816-2002. Journal of Peace Research 44:743-754, 2007.

Maddison A: The world economy: historical statistics. Paris: Organisation for Economic Co-operation and Development (OECD), 2003.

Murray DR, Schaller M: Historical prevalence of infectious diseases within 230 geopolitical regions: A tool for investigating origins of culture. Journal of Cross-Cultural Psychology 41:99-108, 2010.

The_World_Bank: World_Development_Indicators, in. Edited by, <http://data.worldbank.org>, 2011.

World_Health_Organization: Burden of Disease, in Health statistics and health information systems: Disease and injury country estimates. Edited by, Geneva, WHO, 2009, pp. Available at: <http://www.who.int/healthinfo/global_burden_disease/estimates_country/en/index.html>.

World_Health_Organization: MDG 7: Environment sustainability, in Global Health Observatory Data Repository. Edited by, 2011, pp. <http://apps.who.int/ghodata>.
